# Engaging community groups to enhance healthcare access for persons with disabilities in rural Uganda: A qualitative exploration

**DOI:** 10.1371/journal.pgph.0003140

**Published:** 2025-03-31

**Authors:** Andrew Sentoogo Ssemata, Tracey Smythe, Slivesteri Sande, Abdmagidu Menya, Shaffa Hameed, Peter Waiswa, Femke Bannink Mbazzi, Hannah Kuper

**Affiliations:** 1 Disability Research Group, Medical Research Council/ Uganda Virus Research Institute and London School of Hygiene and Tropical Medicine Uganda Research Unit, Entebbe, Uganda; 2 Department of Global Health and Development, London School of Hygiene and Tropical Medicine, London, United Kingdom; 3 International Centre for Evidence in Disability, London School of Hygiene and Tropical Medicine, London, United Kingdom; 4 Division of Physiotherapy, Department of Health and Rehabilitation Sciences, Stellenbosch University, Cape Town, South Africa; 5 School of Public Health, College of Health Sciences, Makerere University, Kampala, Uganda; Erasmus University Rotterdam: Erasmus Universiteit Rotterdam, NETHERLANDS, KINGDOM OF THE

## Abstract

Community participation is a promising strategy for addressing local health needs through identification of context-specific challenges and developing sustainable solutions. However, its feasibility for persons with disabilities, who are often marginalized and excluded from participation, remains uncertain. Our study examines barriers and facilitators to community group participation in improving healthcare access for persons with disabilities in Uganda. Semi-structured interviews with 27 purposively selected persons with disabilities in Luuka district, Eastern Uganda were undertaken between September and November 2022. Questions were asked about participation in existing groups and interest in joining community groups for persons with disabilities to improve healthcare access. All interviews were recorded and transcribed and analysed with a thematic approach. Our study uncovered a notable lack of active engagement among persons with disabilities in existing community groups. Participants expressed a strong desire to belong to disability-focused groups, primarily driven by the desire for unified advocacy. Facilitators for group formation included the opportunity for collaborative problem-solving, unified advocacy, and the chance to share lived experiences. Conversely, barriers to participation encompassed issues such as low self-esteem, financial constraints preventing monetary contributions, and the lack of reasonable accommodations, such as inaccessible meeting venues. Recommendations for group formation included community-wide sensitisation, stakeholder engagement, integration of health-livelihood initiatives, linkage to services, and managing group dynamics to ensure inclusiveness, a manageable group size, and realistic monetary contributions. Persons with disabilities are eager to participate in community groups and recognize the importance of strengthening community-based healthcare initiatives. Addressing barriers to group formation can unlock the potential of these groups to support persons with disabilities effectively. These findings offer valuable insights for developing community-based interventions to enhance healthcare access for persons with disabilities. Further research is essential to fully grasp the key mechanisms and dynamics within these groups to ensure their long-term sustainability.

## Introduction

Approximately 1.3 billion people live with a disability, including around 13% of people living in the African region [1]. Persons with disabilities, on average, have greater general healthcare needs than others in the population due to underlying health conditions, their requirement for assistive devices and other specialist services, and their higher prevalence of risk factors for poor health (e.g., poverty) [2,3]. However, they often experience barriers such as stigma, exclusion, poverty, accessibility and inadequate knowledge and training of providers to accessing routine, as well as specialised healthcare services [4]. Consequently, coverage, affordability and quality of health services are generally worse for persons with disabilities than others in the population, which may contribute to their shorter life expectancy [4]. Addressing these barriers can potentially improve on health outcomes for persons with disabilities and close the life expectancy gap.

The World Health Organisation (WHO) global disability action plan 2014-2021 emphasises the need to remove barriers and improve access to health services and programmes to enable persons with disabilities to fulfil their aspirations in all aspects of life [5]. However, little progress has been made globally in meeting these goals and limited access to health care services remains a major contributor to lower life expectancy and poorer health status, particularly in low-income countries [6,7]. In Uganda, where this study was conducted, the situation is similar to other resource-limited countries. Uganda has approximately 4.5 million persons with disabilities, with the majority residing in rural areas where health care resources are scarce [6,8,9]. Marginalised Ugandans facing barriers to health care, such as transportation, are least likely to afford interventions to address these barriers and experience the highest prohibitive challenges in accessing health centres [10,11].

The WHO recommends the systematic integration of disability into the design, implementation, monitoring and evaluation of all targeted initiatives to support the empowerment of persons with disabilities and overcome these challenges in accessing healthcare [12]. One potential strategy for improving access to health services is using participatory approaches through community-based peer-led health groups to reach and engage persons with disabilities. This approach is recognised as a low cost cross-cutting intervention that can address access barriers to health services at the five access dimensions: approachability; acceptability, availability and accommodation, affordability and appropriateness [13,14].

Evidence is growing that community-led healthcare interventions are effective and affordable solutions to address local healthcare needs. For example, the Participatory Learning and Action (PLA) approach, which involves establishing women’s community groups to identify and implement solutions, has been successful in reducing maternal and new-born deaths by over 20% and is supported by the World Health Organization [15]. In India and Nepal, PLA groups have been cost-effective in reducing neonatal mortality by 24%-30% when supported by government frontline workers and their supervisors, retain a significant contextual importance with a unifying activity and members continue to acquire new knowledge [16,17].

While community groups have been studied for various populations [18,19] and health conditions including TB treatment [20], maternal child health [21–23], sexual and reproductive health [24], and mental health [25]; there has been less attention paid to improving healthcare access overall for persons with disabilities, in the African context. Understanding the feasibility of utilising community groups in improving healthcare access for persons with disabilities is crucial. The effectiveness of these groups can be influenced by contextual factors, which have an impact on health outcomes [17,23]. To develop successful interventions, it is important to understand the perceptions of persons with disabilities in their context. Although community groups can be considered a valuable mechanism for improving health [26,27], such initiatives have not been well adopted for persons with disabilities specifically low resource settings like Uganda, thus the literature in relation to healthcare. This study explored the views of persons with disabilities living in rural Uganda on the perceived barriers and facilitators to participation in community groups, and their recommendations for group formation and organisation, as a strategy to improving access to healthcare.

## Materials and methods

### Study design

This formative qualitative study was undertaken to improve understanding of how to support the co-creation of Participatory Learning and Action for Disability (PLA-D) groups in a rural Ugandan community [28]. We focused on exploring how community groups can be used to generate solutions to overcome barriers to healthcare access and improve healthcare access for persons with disabilities. Luuka district, in Eastern Uganda was chosen purposively as the study area because it has large rural population and is one of the areas in the country with a large number of persons with disabilities [29]. Persons with disabilities in the area also grapple with a number of health challenges and are often excluded from appropriate access to health care services [29].

### Participants and setting

We conducted face-to-face in-depth interviews among persons living with disabilities between 5^th^ September and 26^th^ November 2022. Purposive sampling approach was used to enable researchers to identify participants who could share information-rich narratives of their perspectives towards group formation and participation from the community in Luuka district. Participants included persons with different impairment types including hearing, visual, cognitive, physical, albinism and multiple impairments.

Inclusion of a wide range of different impairment types enabled a comprehensive exploration of participants’ diverse opinions and perspectives. Participants shared reflections about their impairments and suggested forming groups to improve healthcare access tailored to their specific needs. We identified participants from the seven sub-counties and one town council that make up Luuka district.

### Data collection

Data were collected by Ugandan researchers (ASS and SS) who are experienced in qualitative methodology, health and disability research and speak the language spoken in the study area. ASS and SS developed open-ended semi-structured topic guides (S1 Text) based on our key research questions that were used during the interviews. We piloted the guide using the first two interviews to validate it thereby adjusting and rewording some questions based on emerging themes in liaison with the research team. Thereafter, two researchers (ASS and SS) translated the interview guide from English to Lusoga and the research team assessed the guide’s appropriateness and scope allowing for data to be coded thematically.

The first section of the topic guide invited respondents to share their experiences in seeking health care, while the second section explored concepts related to group formation. This provided an opportunity for the exploration of issues related to group dynamics. For example, participants discussed if they were in existing groups and how these groups operated. Interviews for persons with hearing impairment were made accessible and inclusive through the use of a sign language interpreter, who was a member of the research team. For participants with intellectual/ cognitive impairment, we employed strategies such as breaking down complex questions into simpler parts and providing prompts to ensure understanding. Prior to data collection, we tailored our interview guides to be simple, clear, and accessible for all participants, including those with cognitive impairments. Additionally, a caregiver/ family members whom the participant felt comfortable with was present to provide support and clarification, though every effort was made to ensure that the participant’s voice remained central to the conversation. The interviews were conducted in-person at the participants homes, lasted between 40-70 minutes and were recorded, with consent.

### Data management and analysis

The recordings were transcribed verbatim, and the interviews conducted in the local dialect we translated to English language. The transcripts were immediately cross checked against the recordings by one of the co-authors (AM) for completeness, correction of minor transcription errors and to ensure meaning was preserved. All names and any personal identifying information were removed from the transcripts. Two authors (ASS and SS) independently reviewed the transcripts to identify and highlight any emerging themes related to group formation. A thematic qualitative analysis was conducted to using an inductive approach to explore key perspectives linked to group formation. The analysis followed Braun and Clarke [30] six-phase framework: (1) familiarization with the data, (2) generating initial codes, (3) searching for themes, (4) reviewing themes, (5) defining and naming themes, and (6) producing the final report. Thematic saturation was determined when additional interviews no longer produced new information or themes relevant to the research questions. Saturation was continuously monitored throughout data collection, and once no new insights emerged, recruitment was concluded. In total, 27 in-depth interviews were conducted, ensuring diversity in impairment categories and gender distribution to capture a broad range of perspectives. Regular meetings were held to discuss any emerging and divergent themes, and a consensus-driven codebook was developed to support the systematic coding of all transcripts. To ensure rigor, two researchers independently coded a subset of transcripts and later compared their coding to refine definitions and resolve discrepancies. This process enhanced inter-coder reliability and consistency in theme identification. During textual coding, the researchers (ASS and SS) engaged in iterative reading and re-reading of transcripts to identify participants narratives linked to the identified emerging themes. The main themes and sub-themes were refined through continuous comparison across transcripts to ensure coherence and relevance. Data coding was conducted manually using Microsoft Excel. Manual coding was chosen for this study to allow for a more nuanced, in-depth engagement with the data allowing for greater flexibility in capturing emerging themes, particularly given the complexity and diversity of experiences reported by participants across different disability groups. This approach enabled the research team to thoroughly familiarize themselves with the content, context, and subtle meanings in the participants’ responses. Although member checking was not conducted for this study, preliminary results were reviewed and discussed by the research team to address any challenges encountered, refine interpretations, and ensure that the findings accurately represented participants’ perspectives to enhance credibility of the findings. Additionally, investigator triangulation was employed, with three co-authors (AM, SH, FBM,) reviewing the themes to reduce bias and enhance analytical depth. Reflexive discussions were also held to acknowledge and mitigate potential researcher biases.

To enhance transparency, Illustrative participant quotes were included in the results section to exemplify, support, and provide depth to the identified themes. Each quote was clearly attributed to its respective participant category to ensure representation of different viewpoints (e.g., impairment type and gender). To enhance clarity and readability, minor grammatical edits, such as adjusting tense or correcting syntax, were made to some quotes without altering their original meaning or tone. These changes were applied to ensure the authenticity of participants’ voices while making the findings accessible and understandable to a broader audience.

### Ethics statement

The study received institutional and national ethics approvals from Uganda Virus Research Institute (UVRI REC Ref: GC/127/904) and the London School of Hygiene and Tropical Medicine (LSHTM Ref: 26715) and research clearance from the Uganda National Council for Science and Technology (UNCST Ref: SS1348ES) and the Luuka district local government - district health office. All potential participants were provided with written information about the purpose of the study and their rights while participating in the study clearly explained during the consent process. We used simplified information and consent forms for participants with communication challenges and proxy representatives for individuals with cognitive impairment supported the consent process. All participants were above 18 years and provided written informed consent before engagement in any study related activities.

## Results

### Demographic characteristics of participants

We interviewed 27 persons living with disabilities (15 females and 12 males). Each excerpt in this section is labelled with the relevant participant’s impairment category to maintain transparency and ensure that the diversity of perspectives is clear.

Participant demographics are shown in Table 1.

**Table 1:** Participant demographics of interviewed person with disabilities (N=27).

Category	Characteristic	n (%)
Gender	Female	15 (56)
	Male	12 (44)
Impairment	Hearing	6 (22)
	Physical	5 (19)
	Visual	5 (19)
	Intellectual/ Cognitive	5 (19)
	Multiple	5 (19)
	Albinism	1 (4)
Age	Median age (IQR)	31 (21-50) years
Occupation	Formal employment	8 (30)
	Informal employment	15 (56)

#### Membership of persons with disabilities in community groups.

Majority of the participants (n=24) acknowledged existence of community groups and reported not belonging or being actively involved in any existing community groups. Additionally, all participants noted non-existence of community groups specific for persons with disabilities. The other three participants belonged to existing community groups described as Village Savings Loans Associations (VSLAs). In these groups, members met regularly to save money, borrow, and repay loans.

*“There are some village-saving groups I have heard of in this community but there is no one with a disability who goes to them. They say you must have money to save every week to become a member. Most of us are the sole providers in our homes, with very small incomes which also becomes an issue.”* Male, 23 years, Albinism and visual impairment

Other groups that convened collaborated on agricultural projects, meeting with greater frequency than the savings groups. Both entities primarily emphasize livelihood initiatives, yet involvement of persons with disabilities remains minimal or absent.

*“I know there are groups here that work together on agricultural projects. Members support each other and also get government support for example seedlings, animal feeds. I have visited some groups in my capacity as a local leader and none of them has a person with disabilities.”* Female, 43 years, PWD councillor, visual impairment

All the participants interviewed expressed great interest in joining community participatory groups focussed on persons with disabilities. People reported that being a member of a group with persons with different impairments would encourage them to feel that they belong to “one family of persons with disabilities” where they can share life experiences. This would give them hope and encourage them not to feel lonely in the world. The key motivation to join these groups was associated with the ability to save and borrow money especially during times of greatest need - (loss of a loved one, pay medical bills, invest in a small business). Belonging to an organized group was reported to be a source of confidence and hope that they will get the support they desire from health systems as they speak with one voice.

#### Reasons for joining the community groups.

All participants were interested in joining community groups that promote collective action, support, and advocacy in relation to general healthcare. Participants narrated that the groups would give them a strong voice for action and advocacy. They would have an opportunity to share personal experiences, and hoped to receive support, such as startup capital, for entrepreneurship from the groups.

### Platform for problem and solution identification

Most interviewees reported that their main motivator to join a group was the desire to have a uniting platform for joint identification of challenges affecting persons with disabilities. Participants indicated that having an opportunity to share experiences among group members was an easy channel to identify challenges affecting persons with disabilities as well as share success stories to some of their challenges.

*“When there are groups where we can meet together, it is easy to discuss the challenges that we face. It is easy for us to get to know how to handle and solve the problems our own way, and it gives us a way forward on how to handle these challenges.”* Female, 40 years, physical disability.

The collective identification of problems was indicated as an easy opportunity to find solutions and manage challenges. Participants explained that solving problems together reduces the individual burden of handling challenges independently, especially considering already marginalised situations.

*“We have similar problems so rather than pondering over something individually which may be hard for you, someone from the group could share their opinion about the same thing that proved difficult for you and when you take on their advice, your problem is resolved. There is power in being in a group than if you are alone.”* Male, 34 years, physical disability.

### Opportunity for sharing experiences

Persons with disabilities who were interviewed reported that they expected to share their experiences in these groups, which would encourage them to join. Being a member of a group was viewed as an opportunity to freely share their experiences in a safe environment.

*“I am a person with a disability, I think it is good to share experiences in groups in an inclusive way accommodating all our needs. That will be a valuable experience for me.”* Female, 26 years, hearing impairment.

Participants also viewed the groups as providing possibilities to learn and get support from their peers.

*“I would love to be part of that group because I will get support and when we are together in a group, there’s a lot you can learn from each other.”* Female, 23 years, Cognitive impairment.

### Speaking with one voice to demand for services

To some participants, being a member of a community group is an opportunity to have a strong voice to advocate and access healthcare services. Participants indicated that they would join a group so that they can engage communities to address the systemic barriers to healthcare access.

*“I am interested in belonging to a group because when you are in a group and you concentrate on things concerning health, it is possible to have one voice and with that one voice, you can able to get the healthcare worker or two to visit the group, provide care to the group members, talk to them about their health and for me that is important.”* Female, 29 years, multiple disability.

Participants noted that community groups are platforms to advocate for their rights and policy changes, raise awareness about the importance of inclusive healthcare services.

### Using groups to bring healthcare services closer

The desire was expressed to have easier and more convenient access to healthcare services through the groups. Participants indicated that establishment and utilisation of the groups would support bringing services nearer to persons with disabilities, which will encourage them to join the groups. The groups are additionally anticipated to lessen some of the challenges associated with long distances to the health facilities.

*“I would be interested to join the group if they are working towards bringing health services that we are not able to get from extremely far health facilities closer to us. If they say that today, a healthcare worker will be coming to sensitize us and offer some services during these group meetings, it will interest me so much to come and join these meetings.”* Female, 40 years, physical disability.

Additionally, participants believed that establishing partnerships by having healthcare workers participate in group meetings, offering services and education, would encourage collaboration on initiatives aimed at making healthcare access easier and more convenient, thereby increasing their interest in joining these group.

### Empowerment and support

The expectation of economic empowerment, support and development within the formed groups was considered as a facilitator to group formation for the majority of the participants interviewed. The groups are expected to provide encouragement, emotional support, practical advice, economic sensitization and, empowering individuals to navigate the healthcare system.

*“I will join because I know it is going to promote the development for persons with disabilities in the village. We can use the groups to share experiences, resources, and information related to healthcare. We can get people to sensitize us about work, livelihood and how we can be self-sustainable.”* Male, 50 years, Visual impairment.

#### Barriers to group formation and joining community groups.

Low self-esteem among persons with disabilities, the requirement to pay a group membership fee, failure by group members to make monetary contributions, and lack of reasonable accommodation within the groups were described as barriers to group formation.

### Entrenched social exclusion and lack of sensitization

Entrenched social exclusion among persons with disabilities was described as a major barrier for them to take individual actions something that would affect group formation. The low self-esteem together with the lack of community awareness about groups was described as a barrier to group formation.

*“At times, people might not be aware of what is happening within the community, not aware about the group while others might not know what to do.”* Female 45 years, Visual impairment.

Participants expressed little confidence on how to participate in the groups, with fear of discrimination or exclusion which would impact their confidence and willingness to engage in group activities.

*“People with disabilities mostly, have low self-esteem which is the major issue and may even fear to join in the groups developed for them. They need to be sensitized as these groups are meant to help them too.”* Male, 50 years, Visual impairment.

### A fee requirement for group membership

Economic barriers linked to their limited financial resources was noted as a hinderance to group formation and participation. The group membership fees and the routine saving requirements in the existing groups in the communities were indicated as some of the reasons why the majority of persons with disabilities don’t belong to any of the community groups.

*“What would stop me from joining the group is they start by asking for membership fees and for regular savings. I would love to join a group, but I have no ability to pay the mandatory member fees and save in every meeting.”* Female, 40 years, physical disability.

Some participants noted that failure of group members to make monetary contributions was described as a lack of teamwork demotivating other people to belong to such groups.

### Absence of reasonable accommodation

Lack of reasonable accommodation, absence of assistive devices for persons with disabilities to facilitate them to attend group meetings was a reported barrier.

*“Am not encouraged to join a group where there is no reasonable accommodation or resources to support each other. You have someone with a hearing impairment, and we cannot communicate with them or someone in a wheelchair and they cannot reach the meeting venue.”* Male, 34 years, physical disability.

The inadequate provision of reasonable accommodations for the group activities in the community can hinder full participation and inclusion of persons with disabilities hampering their desire to join and participate in the community groups.

#### Recommendations for group formation.

Persons with disabilities suggested various recommendations for the establishment of community groups including sensitization, stakeholders’ involvement, integrating healthcare services within the groups, economic empowerment, and managing group day-to-day dynamics.

### Group awareness and sensitization of persons with disabilities

Persons with disabilities recommended community sensitization about the groups such that people can become aware of the groups, understand their objectives which interest them to join. This would potentially counter the barriers associated with low self-esteem and lack of information.

*“I think the first step would be to bring people together and sensitize them about the need for the group, how they work, why they should join such that they can appreciate the idea and the benefits of such a group”* Male, 40 years, physical impairment.

### Stakeholders’ engagement and group formalisation

Community participation and incorporating stakeholders were suggested as a simple technique to map and locate persons with disabilities. Additionally, participants noted that supporting group registration and formalization within the existing government structures and regulations would strengthen and affirm the members and the group. Group sustainability and ownership was also tied to stakeholder engagement.

*“I think the idea of community groups is ok, we just need to work with our disability organisations, to own the groups, have them registered so they can also benefit from government programmes.”* (Female, 26, student, hearing impairment)

Participants expressed a desire to play a leading role in the group formation, taking ownership of the group’s process and dynamics. This would create an environment that encourages participants to view themselves as key stakeholders and bonafide group members.

### Established organizational structure and managing group dynamics

Central to the group dynamics were ideas related group size, leadership, inclusiveness, and nature of meetings. Participants noted that the groups should be accommodative with a manageable size not to lose the sense of belonging. Groups need to be inclusive of all disabilities and the group leadership needs to be representative of various disabilities. This will avoid dominance of a particular disability, provide opportunity for sharing lived experiences across disabilities and close supervision for easy decision making.

*“We should have a group that is not being too big where we can manage each other, and we are from the same community. These groups need to be for and managed by persons with disabilities because we better understand each other.” Female 25 years, Albinism.*

If there are to be any monetary contributions, it was advised to set them at levels that those with disabilities could afford.

*“If you are asking for membership fees or for savings, put them at a reasonable fee not to discourage members because even the shared resources could bring us together. Let us start small by saving UGX 1000 (approx.$0.3), that may be easy for members to save.”* Male, 23, person with a disability.

### Establish holistic groups

Participants recommended that the groups need to be holistic in nature to cater for a broad range of needs for persons with disabilities rather than problem or need specific. Their expectation is for the groups to be integrative and comprehensive to tackle several needs such as economic empowerment, health, service delivery.

*“The groups should be set up to support our health and income in our homes. Of course, if you’re not healthy you can’t earn.”* Female, 45 years, Physical impairment.

The groups were likened to a bridge to services and support for the members. Participants noted that groups will be a good platform through which health care workers could be invited to share information on health promotion and prevention thereby bring healthcare closer to the communities.

*“The group should be supporting us, giving us help, supporting their members access services from the government. It is particularly good for the group to be able to provide for that makes us very happy”* Male, 23, farmer, Multiple impairment.

## Discussion

Our study found limited participation in existing groups among persons with disabilities, who expressed an ardent desire to join disability-focussed groups primarily for a sense of belonging and to experience shared experiences and support. Key facilitators for group formation included problem-solving platforms, collective advocacy, improved healthcare access shared experiences and economic empowerment. Barriers encompassed low self-esteem, membership fees, financial constraints, and lack of reasonable accommodation. Recommendations included community awareness, stakeholder involvement, integrating health and livelihood initiatives, connecting members to services, improving healthcare accessibility, and effective management of group dynamics to ensure inclusivity and effective decision-making.

To maximise the benefits of the groups, there is a need to attend to the identified barriers that emerge in groups formation and operation which, left unchecked, may limit the potential outcomes of the group in improving access to healthcare and threaten their long-term sustainability [23]. Common challenges such as difficulties in ensuring equal participation, disparities in group dynamics, such as power imbalances or lack of clear leadership, can hinder effective collaboration and decision-making. By proactively identifying and addressing such challenges makes community groups more inclusive, equitable, and effective in generating solutions whilst enhancing the group’s success over time [16,27].

From the findings, the motivations of participants for joining disability groups were primarily driven by a need for social connection and shared experiences, economic empowerment, and mutual support. The groups were seen as an opportunity to share challenges, experiences and solutions to improve their health and wellbeing. We found that community groups constitute a fundamental part of the participatory process to promote collaboration between participants, incorporate different perspectives, and guarantee community change such as equity-focused public health actions [31]. Important to note is that whilst persons with disabilities are hoping for economic empowerment and financial contribution through the community groups, a requirement of a mandatory contribution to join the groups created complexities for many economically inactive persons with disabilities as reported previously [32–34].

There are diverse types of groups and group meetings, which serve diverse functions within communities. For example, groups may be designed to meet specific needs. Saving groups in combination with participatory action research have been reported to improve birth preparedness and maternal and newborn health [35]. Some groups are designed to facilitate the sharing of information and foster connections among participants as recommended in this study. These gatherings often serve as platforms for knowledge exchange and social networking, which may contribute to wider health and well-being [36]. Others focus on practical changes, such as savings and investment groups, where members come together to pool resources, save money, or engage in agricultural activities to improve their livelihoods [27]. For example, these groups in rural Eastern Uganda provided social safety nets for communities, facilitated the community mobilization through the regular meetings and other development activities, such as health or literacy training and financial transactions [27]. This is similar to what participants reported in our study. Additionally, there are groups aimed at raising awareness about specific issues, whether they be health-related, social, or environmental such as improved maternal health through the process of establishing and undertaking community groups [37]. These awareness groups often work to educate the community and drive positive change. Participants acknowledged that participation in community groups builds resilient, self-determining communities capable of dealing with complex rural access and equity challenges as reported in previous studies [38,39]. The choice of group type and function typically depends on the specific needs and objectives of the community or individuals involved. For example, in rural Uganda, community antiretroviral groups have been adopted as one of the innovative and efficient differentiated service delivery models tailored to the specific needs to reaching persons living with HIV in the community [40].

From the study findings, we acknowledge that community groups serve as a valuable platform for supporting intervention delivery and behavioural change tailored to the needs of persons with disabilities thereby contributing to the individuals and groups’ overall well-being [36].

Groups can serve as a powerful basis for problem identification and solution generation to improve access to healthcare for persons with disabilities with a relevant and sustainable mechanism. Through collective participation, individuals are able to share their diverse experiences and insights, helping to highlight common barriers and challenges that may not be evident in individual settings. This collaborative environment fosters innovative thinking and the co-creation of solutions that are both relevant and tailored to the unique needs of persons with disabilities [31]. The groups can develop sustainable mechanisms that ensure long-term improvements in accessibility, quality of care, and inclusion within healthcare systems. By harnessing the collective strength of community groups, persons with disabilities can achieve greater health and wellbeing and lead fulfilling lives within their communities.

### Strengths and limitations

Our study has strengths and limitations to consider. The study used purposive sampling to include a diverse range of participants with different impairment types, allowing for a comprehensive exploration of perspectives and recommendations related to group formation and healthcare access. Additionally, the use of in-depth interviews conducted by experienced researchers allowed for the collection of rich and detailed narratives, providing a deeper understanding of the lived experiences of persons with disabilities in the community. However, there are some limitations to consider. As a qualitative study, the sample size may be small, which could limit the generalisability of the findings to other contexts. However, the ability to collect perspectives from participants with various impairments in the community provided in-depth perspectives on group formation. Furthermore, while purposive sampling allowed for a comprehensive exploration, it might introduce some selection bias, as participants were chosen based on specific criteria.

### Implications for research and service provision

Our ambition is to set up community groups of persons with disabilities to identify barriers and generate low-resource community driven solutions to overcome the barriers in accessing healthcare [28]. Persons with disabilities reported a key motivation to join these groups as primarily being associated with the ability to address other needs beyond healthcare such as opportunities to save and borrow money during times of need, which included medical expenses or starting small businesses. Additionally, participants viewed these groups as a mean to identify shared challenges, advocate for healthcare access, provide a closer link to healthcare services, and foster the sharing of experiences among members.

We believe that the concept of community based participatory learning action groups can be applied to inform the design of interventions to improve access to health services for persons with disabilities [28]. Further research is needed to evaluate sustainability of these groups in the community and using these groups to address other unmet needs for persons with disabilities, i.e., education, livelihood, and water sanitation and hygiene initiatives. We recommend the participatory approach to ensure the perspectives of persons with disabilities shape implementation efforts. By gaining insights from the perspectives of persons with disabilities themselves, we can strengthen the development of effective community-based healthcare interventions. The evolution of community group modalities for persons with disabilities must be continuously explored and refined to optimize their potential in enhancing access to health care services. As we raise awareness of the factors that influence successful group formation and participation, we take a significant step towards fostering resilient, self-determined communities capable of overcoming access barriers and promoting equitable health outcomes.

## Conclusion

Groups for persons with disabilities hold promise as sustainable approaches to addressing a wide range of health challenges faced by persons with disabilities. This study sheds light on the critical role of understanding facilitators and barriers to group formation, emphasizing the need for well-managed and inclusive group dynamics. By prioritising the voices and experiences of persons with disabilities, we can pave the way for transformative changes that will benefit the health and well-being of the entire community.

## Supporting information

S1 Text Interview guide. (PDF)
